# Editorial: Gestational diabetes: where are we and where are we going?

**DOI:** 10.3389/fcdhc.2024.1518345

**Published:** 2024-11-22

**Authors:** Federica Piani, Giovanni Tossetta

**Affiliations:** ^1^ Hypertension and Cardiovascular Risk Research Center, Medical and Surgical Sciences Department, Alma Mater Studiorum University of Bologna, Bologna, Italy; ^2^ Department of Experimental and Clinical Medicine, Università Politecnica delle Marche, Ancona, Italy

**Keywords:** gestational diabetes mellitus, GDM, pregnancy, complications, placenta, inflammation

## 1 Introduction

Gestational diabetes mellitus (GDM) is a complex and increasingly prevalent condition that poses significant challenges to maternal and fetal health, as well as public healthcare costs. In this Research Topic, we present a collection of studies highlighting innovative approaches to GDM management. These include the use of technology, such as integrated bioinformatics analysis and data-driven clusters, and insights into GDM pathophysiology and risk factors.

## 2 Gestational diabetes epidemiology and risk factors

GDM is defined as glucose intolerance that develops during pregnancy, typically diagnosed during the second or third trimester. The current prevalence of GDM in Europe is estimated to be around 10.9% based on recent meta-analysis of prevalence studies (1). Rates of GDM are rising globally, partly explained by the increasing obesity and maternal age. International association of diabetes and pregnancy study groups recommendations on the diagnosis and classification of hyperglycemia in pregnancy (IADPSG) suggest as diagnostic criteria: fasting plasma glucose (FPG) ≥5.1 mmol/L (92 mg/dL); 1-hour plasma glucose of ≥10.0 mmol/L (180 mg/dL) and/or 2-hour plasma glucose of ≥8.5 mmol/L (153 mg/dL) during a 75 g oral glucose tolerance test (2).

Known risk factors for the development of GDM are obesity, advanced maternal age, family history of type 2 diabetes mellitus (T2DM), and polycystic ovary syndrome, with other risk factors under study (3). Interestingly, in the study published in the present Research Topic by Hui et al., the highest quartile of liver function index (LFI) was linked to a heightened risk of GDM, with odds ratios (OR) ranging from 1.29 to 3.15. Additionally, a noteworthy interaction between AST/ALT levels and triglycerides (TG) was identified regarding GDM risk (P interaction = 0.026). Interestingly, TG have also been associated with vascular dysfunction, with TG being a possible common pathophysiologic mechanism between GDM and hypertensive disorders of pregnancy (4). As Authors reported, other studies found heterogenous results on different liver function markers, with significant associations of GDM with GGT and liver function index but not with AST and ALT. To overcome possible reverse causation, Authors also performed a Mendelian randomization analysis, showing a causal relationship between ALT levels and GDM with an OR of 1.28 (95% CI: 1.05-1.54). The association between liver function and metabolic dysfunction leading to GDM is crucial not only for developing new risk scores but also for better understanding the pathophysiology of GDM.

## 3 Gestational diabetes pathophysiology: the immune theory

The etiopathogenesis of GDM is unclear, but studies suggest maternal immune dysfunction and chronic inflammation as key contributors (5, 6). Elevated cytotoxic NK cells and dysregulated Tregs and Th17 cells, alongside cytokines like IL-6, IL-1β, and TNF-α, exacerbate insulin resistance, contributing to GDM development (5–8). Inflammation plays also a key role in endothelial dysfunction (9–12) contributing to angiogenic imbalance and the occurrence of cardiovascular diseases in GDM patients (13–15). However, understanding of the immune microenvironment in GDM remains limited (7). Chen et al. in the present Research Topic performed a comprehensive bioinformatic analysis on gene expression profiles of two databases on Human Umbilical Vein Endothelial Cells (HUVEC) RNA-seq data from patients with GDM and controls. Furthermore, Authors evaluated the associations between the six hub of Differentially expressed genes (DEG) with high diagnostic value for GDM and immune cells. The two biomarkers found to have the highest diagnostic value, PLAUR and SLIT2, had a strong correlation with B cells naïve and T cells follicular helper, respectively. The authors emphasize the need for animal studies to validate their findings. In this context, another article published within the same Research Topic examined NK cell functionality in animal models of diabetes by (Xion et al.). Decidual NK cells differ in phenotype and function from circulating NK cells (16, 17). They interact with the fetus by engaging human leukocyte antigen (HLA) ligands expressed on extravillous trophoblasts, promoting immune tolerance between the mother and fetus. In the streptozotocin-induced model of GDM, hyperglycemia disrupted immune homeostasis, affecting both the proportions and functions of NK cells. Authors also analyzed circulating NK cells in women with GDM vs. controls showing that there are no differences in the percentage of total NK cells but there are significant differences in the amount of single NK phenotypes. In summary, the two abovementioned studies submitted in this Research Topic contribute to the growing body of literature on the immune mechanisms at the basis of GDM pathophysiology, providing valuable insights into the cell types and molecular mechanisms involved.

## 4 Pregnancy outcomes and persistence of glucose intolerance

Women diagnosed with GDM face increased risks of complications, including preeclampsia, cesarean delivery, and the likelihood of developing T2D later in life. Indeed, GDM and preeclampsia share several pathophysiological pathways and biomarkers (3, 18). Furthermore, GDM can adversely affect fetal development, leading to higher rates of macrosomia, neonatal hypoglycemia, and long-term metabolic issues for the child (19, 20). Approximately one-third of women diagnosed with GDM using pre-IADPSG criteria will exhibit glucose levels indicative of diabetes or prediabetes during postpartum assessments within 3 months from delivery. Lesniara-Stachon et al. highlighted a crucial point regarding the distinction between diabetes induced by reduced insulin levels and that caused by peripheral insulin resistance. This differentiation is significant for understanding the underlying mechanisms of diabetes and guiding its management and therapeutic strategies. Interestingly, Authors found that predictors of 1-year postpartum glucose intolerance were different among diabetes phenotypes. For women belonging to the insulin resistant cluster, HOMA-IR was the best predictor (OR 1.9). On the other hand, history of GDM (OR 8.7) and FBG (7.8) were the best predictors of 1-year postpartum glucose intolerance in women within the insulin resistant cluster. A precision medicine approach to GDM that considers pathophysiologic subtypes could enhance diagnosis, risk assessment, and treatment strategies.

## 5 Future perspectives

Personalized medicine, allowing for recognition of specific phenotypes with consequent tailored treatment, long term follow-up strategies to reduce the risk of developing T2D and cardiovascular diseases, and translational research to better clarify GDM inflammatory pathogenetic pathways are the main areas to be addressed in future research on GDM. Addressing these research areas would enhance understanding but also improve prevention, diagnosis, and treatment strategies for this increasingly prevalent condition.
